# Forgotten muscle: diaphragm and sudden infant death syndrome

**DOI:** 10.1007/s12519-026-01026-5

**Published:** 2026-04-09

**Authors:** Pontus M. Siren

**Affiliations:** Inependent Researcher, Crassier, Switzerland

Contemporary sudden infant death syndrome (SIDS) research is at an impasse. After decades of effort and thousands of scholarly publications, there is no consensus on the underlying mechanism or coherent explanation for how diverse and seemingly unrelated risk factors contribute to the syndrome. Equally perplexing is our inability to explain the cause of death in SIDS. While the underlying mechanism is unresolved, compelling evidence suggests that the syndrome likely has a respiratory origin. For the past three decades, SIDS research has focused on the central nervous system (CNS) and on how dysfunctional homeostatic mechanisms controlling arousal, cardiorespiratory function, and neurotransmission may contribute to respiratory failure [1]. However, despite significant and ongoing efforts, this approach has failed to provide conclusive answers to the SIDS enigma. Given the likely respiratory origin of the syndrome, it is surprising that the diaphragm—a vital ventilatory muscle—has been overlooked in SIDS research [2]. To foster an informed and critical debate regarding its possible role in the syndrome, the editorial examines the diaphragm’s significance in ventilation and considers its vulnerabilities and failure mechanisms in the context of SIDS and its risk factors.

Approximately 13,300 scientific articles on SIDS were indexed in PubMed between 1960 and January 2026. This extensive body of research has explored the possible role of all cardiorespiratory organs in SIDS, with one notable exception. During the same period, only 63 articles on “SIDS and diaphragm” were published. No clinical studies or trials, meta-analyses, or reviews are indexed. Typically, the diaphragm is not included in the pathological investigation of suspected SIDS cases [3, 4].

Most of the articles do not examine the diaphragm’s role in the syndrome or do so only tangentially [5–22]. The pathological research and observational studies do not support the exclusion of the diaphragm as a possible contributing factor in SIDS. The same is true for animal studies [23–41]. None of the articles presenting novel SIDS hypotheses or letters to the editor argue for its exclusion [42–53]. Six publications highlighted significant pathological abnormalities in the diaphragms of affected infants. Three studies reported that SIDS cases have fewer type I (fatigue-resistant) diaphragm muscle fibers than controls [54–56]. The authors postulated that this may increase the risk of diaphragm fatigue, a hypothesis that is consistent with animal data showing that newborns with a low proportion of type I fibers are susceptible to fatigue [57]. Pathology of diaphragmatic fibers from 242 SIDS victims shows acute and recent muscle fiber necrosis accompanied by hemorrhages and edema in 82% of the samples [58], which is consistent with persistent ventilatory effort under hypoxic conditions [59]. Silver and colleagues noted that diaphragmatic contraction band necrosis is characteristic of acute asphyxia and is prevalent in birth asphyxia and SIDS (11 of 26 and 19 of 30 cases, respectively) [60]. Consistent with these results, diaphragm muscle fiber ruptures and contraction bands were found in the SIDS infants and the control group, who died of asphyxia, strangulation, drowning, pneumonia, and undetermined causes [61]. The key parameters and results of the pathological studies are summarized in Supplementary Table 1. The evidence on the possible role of the diaphragm in SIDS is dated and limited in scope and scale. These data do not support the exclusion of the diaphragm as a possible contributing factor to the syndrome; rather, there is preliminary evidence of structural and pathological abnormalities in SIDS diaphragms.

## Respiratory failure in sudden infant death syndrome

It is generally accepted that acute non-traumatic death results from either respiratory or cardiac failure. The possible role of heart dysfunction has been comprehensively investigated, but the evidence does not support a cardiac etiology for SIDS [62]. Rather, as Kinney and Thach argue, a large body of research—ranging from pathological and clinical data to heart rate and respiratory analyses of SIDS cases—suggests that the syndrome has a respiratory origin [1].

The respiratory system consists of two components: the gas-exchanging organ (i.e., the lungs) and the ventilatory neuromuscular pump (hereafter the ventilatory pump), which ventilates the lungs. The ventilatory pump comprises the chest wall, ventilatory muscles, respiratory controllers in the CNS, and the spinal and peripheral neural pathways between the CNS and the muscles. Respiratory failure occurs due to either lung failure, which presents as hypoxemia with normocapnia or hypocapnia (type I respiratory failure), or ventilatory pump failure characterized by alveolar hypoventilation and hypercapnia (type II or hypercapnic ventilatory failure). While hypoxemia characterizes both conditions, the key distinguishing feature of ventilatory pump failure is elevated pCO_2_ [63].

There is compelling evidence of both hypoxia and hypercapnia in SIDS [1, 64, 65], suggesting that the syndrome is caused by dysfunction of the ventilatory pump. Kinney and Thach argued that SIDS has a respiratory pathway that begins hours or days prior to death and, as such, is progressive rather than sudden. Infants first experience asphyxia and brain hypoperfusion, or both, without arousal. This is followed by increasing hypoxia and hypercapnia, loss of consciousness, hypoxic coma, extreme bradycardia, hypoxic gasping, sustained apnea, and eventual death. There is evidence of intermittent and chronic hypoxia in SIDS [1].

Acute hypercapnic respiratory failure has three principal causes: (1) dysfunction of the CNS; (2) impairment of neuromuscular transmission; and (3) fatigue of the respiratory muscles. These failure mechanisms—central, transmission, and peripheral—can occur independently or synergistically, and manifest similar clinical symptoms [63]. The intensive investigation of the central and connective nervous system has not yielded definitive answers, nor has research identified consistent evidence of genetic abnormalities, trauma, deformities, or diseases of the nerves, rib cage, or muscles in SIDS cases [66]. The third failure mechanism, critical fatigue of the respiratory muscles, has been ignored by contemporary SIDS research.

## Diaphragm, the vital ventilatory muscle

The diaphragm is the primary ventilatory muscle in mammals, and in human adults, it contributes approximately 80% of the inspired air volume during regular breathing [67]. Young infants have underdeveloped secondary ventilatory muscles and rely primarily on diaphragmatic breathing [68]. Functional compromise of the diaphragm leads to the cessation of independent breathing, the termination of effective ventilation, and failure of tissue respiration [69, 70]. Given its vital role, the diaphragm has evolved to be highly resistant to terminal fatigue, and in adults, functional failure is rare absent significant underlying morbidity [59]. Severe clinical conditions, such as acute or chronic infection, can cause an imbalance between ventilatory muscle energy supply and demand, leading to ventilatory muscle fatigue characterized by a loss of force output, inadequate inspiratory pressure, decreased minute ventilation, hypoxia, and hypercapnia [63]. If uninterrupted, progressive diaphragm fatigue can lead to respiratory failure and death [71].

Diaphragm failure is uncommon in healthy adults and children alike. However, muscle strength and endurance are functions of mass, fiber composition, and contractile properties [72], and infants aged ≤ 6 months are vulnerable to clinically significant diaphragm fatigue absent serious disease. Dassios and colleagues emphasize that several physiological factors increase the risk of diaphragm fatigue and ventilatory pump impairment in young, and especially in premature and low-birth-weight (LBW), infants [69].

1. Neonates have low diaphragm and accessory muscle mass. The infant diaphragm is small in size, and in preterm infants [gestational age (GA): 26–32 weeks], only 1.09 ± 0.08 to 1.74 ± 0.04 mm thick [73]. An increase in diaphragm size directly correlates with inspiratory force, and weaker ventilatory muscles require more energy (relative to their maximum capacity) to perform a given amount of work. Maximal inspiratory force increases with GA [69], and both maximal inspiratory and expiratory pressures during crying are higher in term compared to preterm infants [74].
2. Young infants have a structurally immature diaphragm. Premature infants (GA < 37 weeks) have only 9.7% fatigue-resistant muscle fibers, compared with 25% in full-term infants [75]. The proportion of type I fibers correlates directly with fatigue resistance of the diaphragm, and before reaching structural maturity, infants can sustain only a small percentage of the maximum transdiaphragmatic pressure before exhibiting signs of muscle fatigue [69, 76].
3. The ribcage of newborns consists primarily of cartilaginous tissue rendering the chest wall approximately three times as compliant as the lungs. The compliant chest wall offers less resistance to the lungs’ inward recoil causing the relaxation volume to be reduced to only 10%–15% of total lung capacity, compared to 30%–35% in adults. This decreases the mechanical efficiency of respiration [68, 77, 78].
4. In neonates, the diaphragm is flattened and the ribs are positioned horizontally, placing the respiratory muscles at a mechanical disadvantage and impairing ventilatory efficiency [68, 79, 80].
5. The low oxidative capacity of the diaphragm in young infants renders it susceptible to fatigue [68, 81].

The GA is the main determinant of diaphragm structural and functional maturity [68], and these vulnerabilities are pronounced in preterm infants, who have a greater risk of diaphragmatic fatigue, especially when the respiratory load is increased [69]. However, the elevated risk of fatigue is transitory, as the diaphragm, secondary respiratory muscles, and rib cage mature rapidly during the first months of life. Infants display adult levels of maximal transdiaphragmatic pressure by 6 months of age [77, 82], and the proportion of type I fibers in term infants increases progressively to approximately 40% by 3 months of age and reaches adult levels of 50%–55% by 7–8 months [75]. In context, approximately 95% of SIDS cases occur in infants aged 6 months and younger [83], preterm infants have a fourfold greater risk of SIDS than full-term neonates do, and their risk for SIDS is inversely correlated with GA [84].

## Impact of SIDS risk factors on diaphragm function

To further inform the debate, this paper considers how select SIDS risk factors impact diaphragm function. The role of nonlethal infections in the syndrome has been extensively investigated, and a significant share of SIDS cases, up to 80%, shows evidence of clinical or sub-clinical infections [85, 86]. Ferrante and Opdal note that a large proportion of SIDS victims have elevated levels of interleukin-6 (IL-6) in their cerebrospinal fluid, and cases with high IL-6 levels also have increased expression of both immunoglobulin (Ig) A and human leukocyte antigen-DR in the laryngeal mucosa. Elevated IgM and IgG immunocytes have also been reported in SIDS patients compared with controls [87]. Early infancy is “a time of infection”, and the presence of cytokines and bacterial and viral pathogens is not surprising. However, while there is no consensus on their role, a large body of clinical, observational, and pathological evidence suggests that nonlethal infections play a salient role in this syndrome [88].

Diverse nonlethal infections, both viral and bacterial, can acutely and materially impair the diaphragm's force-generating capacity [49, 89]. Supinski and Callahan emphasized that “studies in both humans and animals indicate that even minor infections can induce significant reductions in respiratory muscle strength” [90]. Infections increase mitochondrial free radical generation [91], decrease mitochondrial function, activate proteolytic pathways, and reduce contractile protein function in the diaphragm, resulting in a rapid reduction in diaphragm force generation capacity [92]. In humans, nonlethal infections can impair diaphragm function within 1–48 hours [93–95], and animal models have shown that infections can reduce diaphragm force generation capacity by 50% in 24 hours [96, 97] and by 80% in 48 hours [98]. Infections that induce ventilatory muscle weakness do not compromise the cardiac muscle [99].

Non-lethal infections impair diaphragm function, but it is important to emphasize that they alone do not, by definition, cause SIDS. Rather, they seem to be contributing factors that, together with other risk factors, increase the risk of SIDS. This synchronistic effect is illustrated by the interplay of the prone sleeping position and nonlethal infections. Both are associated with increased SIDS risk, and both negatively affect diaphragm function by either decreasing its force-generating capacity (infection) or increasing its workload (prone position). The prone position shortens the diaphragm and is associated with a 15%–30% increase in lung volume. In adults, a similar increase results in a 40%–50% reduction in diaphragm strength and endurance [100–102]. Notably, the risk associated with the prone sleeping position differs between infants with detectable infections and healthy subjects. Two large and geographically disparate epidemiological studies have shown that the risk associated with the prone position is significantly greater in infants with confirmed infections. Ponsonby and colleagues analyzed data from 58 SIDS cases and 120 controls in Australia and reported a tenfold higher SIDS risk in infected infants but only a slightly increased risk in the uninfected cohort [103]. A large Nordic study reported a 29-fold higher SIDS risk in infants with detectable infections than in the uninfected cohort [104]. These data show that two seemingly unrelated factors that impair diaphragm function can synergistically increase the risk of SIDS.

Sleep is another important risk factor for SIDS, and the original name of the syndrome—“cot death”—underscores its association with the syndrome. Considering that neonates sleep 16–18 hours per day, the temporal association is not surprising. However, a study of 325 SIDS cases reported that 83% of deaths occurred during nighttime sleep, mostly within a relatively narrow time window between approximately 00:00 and 05:00 [105]. The sleep state of these infants was not determined, but another study revealed that, compared with controls, SIDS patients spent more time in rapid eye movement (REM) sleep between 03:00 and 07:00 [106]. A study on vulnerable infants who were resuscitated from a potentially life-threatening ventilatory event also reported a significant increase in REM sleep between 02:00 and 05:00 [107]. There is no consensus explanation for the link between SIDS and sleep [108].

During REM sleep, the intercostal muscles—which stabilize the highly compliant infant chest wall and optimize diaphragmatic length for efficient ventilation—are largely or entirely inhibited. The phasic and tonic inhibition increases diaphragmatic workload, decreases the mechanical efficiency of ventilation, and results in paradoxical motion of the ribcage, chest wall distortion, decreased tidal and end-expiratory lung volumes, and reduced transcutaneous oxygen partial pressure [69, 109, 110]. In adults, observable diaphragm weakness and hypoventilation typically first appear during sleep, and paradoxical breathing is indicative of severe diaphragm weakness. The risk of hypoxemia and hypercapnia is elevated, particularly during REM sleep [111, 112]. Nocturnal oxygenation is correlated with diaphragm strength, and during REM sleep, episodic oxygen desaturation and central hypopnea are caused primarily by reduced activity of the accessory ventilatory muscles [113]. Paradoxical breathing is a sign of inefficient ventilation also in healthy preterm infants [114, 115], but it decreases with age, possibly reflecting the maturation of the ventilatory muscles and the chest wall [116].

Evidence from healthy full- and preterm infants, SIDS victims, and near-miss cases shows that REM sleep can significantly increase diaphragmatic workload and the risk of muscle fatigue: (1) in young infants, rib cage motion in REM sleep is nearly exclusively paradoxical and is especially pronounced in preterm infants who already display signs of ventilatory distress [117]; (2) neonates show marked inward movement of the rib cage in REM sleep, accompanied by a 150% increase in diaphragmatic electromyography (EMG) amplitude, indicating a significant increase in respiratory workload [118]; (3) full-term infants have an 18% higher ventilatory rate in REM than non-REM (NREM) sleep, evidence of increased diaphragm workload [119]; (4) in newborns, ventilatory muscle fatigue patterns appear only in REM sleep, together with markedly reduced intercostal activity, rib cage retraction, hypoventilation, and hypercapnia [115]; and (5) SIDS infants on electrocardiogram and respiratory monitors had higher heart rates and increased chest wall compliance than controls in REM sleep, but not in NREM sleep. REM sleep was characterized by low oxygen reserves and apnea, which rapidly lead to hypoxemia and tachycardia [120].

A study on LBW infants (mean birth weight 1251 ± 424 g) who experienced 66 episodes of ventilatory muscle fatigue highlighted the interplay between intercostal muscle inhibition and respiratory distress. The infants were monitored by diaphragmatic EMG frequency spectrum analysis and responded to fatigue in two ways: 5 of the 12 infants who displayed muscle fatigue consistently recruited the intercostal muscles to stabilize the chest wall and normalize the diaphragmatic frequency spectrum. However, seven infants did not recruit the intercostals and first experienced severe diaphragmatic weakness, followed by apnea and, in some cases, bradycardia. This group required stimulation and substantial ventilatory support (O_2_ at 30%–70%) to prevent clinical deterioration. The authors noted that REM sleep inhibits the intercostal muscles and “dramatically increases” the diaphragmatic workload in young infants. They postulated that the diaphragm fatigue and clinical deterioration observed in the study were precipitated by intercostal muscle inhibition associated with REM sleep [121].

Like infections, REM sleep, and the prone position, hypoxia is strongly associated with SIDS [1]. The diaphragm is highly oxidative and vulnerable to hypoxic insults [122]. Hypoxia can initiate a negative feedback loop in which impaired oxygenation reduces diaphragm performance, which further compromises muscle oxygenation and function [45]. The hallmarks of sustained hypoxia are reduced force-generating capacity and compromised ability to cope with increased workloads [123]. Studies in humans and animals have shown that hypoxia reduces the peak force-generating capacity of the diaphragm by 20%–25% and 30%, respectively [124, 125].

Hypercapnia is similarly associated with SIDS, but its specific role in the syndrome is unclear. Diaphragm dysfunction causes alveolar hypoventilation, leading to elevated pCO_2_. This stimulates the CNS to increase minute ventilation, adding to the diaphragm’s workload and increasing the risk of fatigue [126, 127]. Animal models show that hypercapnia also depresses the diaphragm’s force-generating capacity, and can contribute to a negative feedback loop where diaphragm fatigue and hypercapnia synergistically impair diaphragm function, thereby increasing the risk of both [72].

A detailed discussion of all known SIDS risk factors is beyond the scope of this article. However, this and prior publications have examined the effects of 15 diverse SIDS risk factors on diaphragm function, each of which increases the risk of diaphragm fatigue, either by increasing the workload or by reducing the force generation capacity [2, 43–45, 47, 49]. The combined effect of multiple concurrently arising risk factors on diaphragm function has not been modeled or clinically investigated.

## Future directions

The diaphragm is a vital ventilatory muscle that has been overlooked in SIDS research. Infants six months and younger—particularly LBW infants and those born preterm—are susceptible to diaphragm fatigue and poorly tolerate increases in ventilatory workload. As McCool and Tzelepis emphasized, the combination of diaphragmatic weakness and any process that increases the work of breathing may overwhelm the capacity of even a mildly weakened diaphragm [128]. SIDS infants typically present with multiple risk factors that impair diaphragm function or increase its workload: nonlethal infections and hypoxia can reduce the diaphragm’s force-generating capacity by 50%–80% and 20%–30%, respectively; the prone position is associated with a significant reduction in diaphragm strength and endurance and REM sleep with an 18% increase in diaphragmatic work. The combined impact of these and other SIDS risk factors on diaphragm function in vulnerable infants should be investigated.

Clinical and animal protocols involving inspiratory resistive loading (IRL) may be relevant for investigating the impact of SIDS risk factors on diaphragm function. When preterm infants are exposed to loaded breathing, they experience a 50% decrease in minute ventilation and tidal volume compared with controls. If the load is sustained, the risk of diaphragmatic fatigue increases [129, 130]. An IRL study in rats revealed that when the diaphragm is subjected to excessive contractile demands, the respiratory pump eventually fails because of inadequate pressure generation. When the IRL is applied in stages, diaphragm fibers are damaged, and diaphragmatic fatigue occurs together with increased central drive. Progressive IRL-induced respiratory distress is consistent with peripheral, rather than central, failure [131].

Dassios and colleagues provide a comprehensive and current review of the methods for the assessment of respiratory muscle function [69]. These include EMG, respiratory pressure and composite indices, maximal respiratory pressures, phrenic nerve stimulation, the tension time index of the diaphragm, thoracoabdominal asynchrony, the relaxation rate of the respiratory muscles, and diaphragmatic ultrasound. Each has strengths, limitations, and specific clinical applications that the authors discuss in detail. Point-of-care ultrasound is emerging as a popular non-invasive clinical tool that enables real-time evaluation of diaphragmatic function. Importantly, normative data have been established for healthy term infants. However, meaningful challenges, such as access to skilled operators who can accurately image and interpret data and the lack of standardized clinical protocols and decision algorithms specific to neonates, still exist [132]. Despite these challenges, contemporary diagnostic methodologies have step-changed the clinical assessment of diaphragm function in at-risk infants, and compared with the postmortem pathological studies of the 1980s and 1990s, they represent a significant improvement and clinical opportunity.

The diaphragm can fail when subjected to excessive ventilatory demand. Anatomical, physiological, and pathophysiological factors predispose young infants—particularly preterm and LBW neonates—to ventilatory muscle impairment and fatigue and, in some circumstances, respiratory failure. Fifteen diverse and seemingly unrelated SIDS risk factors increase the risk of diaphragm fatigue by either increasing the workload or decreasing the force-generating capacity of the diaphragm. The diaphragm, secondary respiratory muscles, and rib cage mature rapidly, and infants reach adult levels of fatigue resistance and force generation by approximately 6 months of age. This editorial aims to stimulate a critical debate regarding the potential role of the diaphragm in the syndrome and encourages readers to reconsider its exclusion from contemporary SIDS research.

## Supplementary Information

Below is the link to the electronic supplementary material.

Supplementary file 1 (XLSX 14 KB)

## Data Availability

Not applicable.
